# Tartary Buckwheat Bran: A Review of Its Chemical Composition, Processing Methods and Food Uses

**DOI:** 10.3390/plants12101965

**Published:** 2023-05-12

**Authors:** Takahiro Noda, Koji Ishiguro, Tatsuro Suzuki, Toshikazu Morishita

**Affiliations:** 1 Hokkaido Agricultural Research Center, National Agriculture and Food Research Organization, Shinsei, Memuro, Kasai-gun 082-0081, Japan; 2 Kyushu-Okinawa Agricultural Research Center, National Agriculture and Food Research Organization, Suya, Koshi, Kumamoto 861-1192, Japan

**Keywords:** Tartary buckwheat, bran, functional compound, rutin, food

## Abstract

Tartary buckwheat (*Fagopyrum tataricum* Gaertn.) containing large amounts of functional compounds with antioxidant activity, such as rutin, has attracted substantial research attention due to its industrial applications. Particularly, the functional compounds in Tartary buckwheat bran, an unexploited byproduct of the buckwheat flour milling process, are more concentrated than those in Tartary buckwheat flour. Thus, Tartary buckwheat bran is deemed to be a potential material for making functional foods. However, a review that comprehensively summarizes the research on Tartary buckwheat bran is lacking. Therefore, we highlighted current studies on the chemical composition of Tartary buckwheat bran. Moreover, the processing method and food uses of Tartary buckwheat bran are also discussed.

## 1. Introduction

Buckwheat is characterized as a pseudo-cereal due to its cereal-like characteristics, belonging to the genus *Fagopyrum* in the family Polygonaceaea in the order Caryophyllales. Among buckwheat species, two species, namely, common buckwheat (*Fagopyrum esculentum* Moench) and Tartary buckwheat (*Fagopyrum tataricum* Gaertn.), are widely known. Tartary buckwheat is a traditional crop that is cultivated and utilized mainly in Asian countries, including Japan [1]. Tartary buckwheat has a nutritionally excellent profile with higher levels of protein alongside balanced amino acids, dietary fiber, vitamins, and minerals [1,2]. Additionally, it has attracted increasing attention because of its higher levels of functional compounds with antioxidant activity, such as flavonoids: a group of polyphenolic compounds [1,2]. The major functional component of Tartary buckwheat is rutin: a flavonol glucoside composed of quercetin and rutinose [3]. Tartary buckwheat seeds have been shown to contain approximately 100-fold higher levels of rutin than common buckwheat seeds [3,4]. Rutin exhibits several health benefits, such as strengthening human capillary fragility [5], anti-hypertensive properties [6], anti-inflammatory properties [7], antioxidant properties [4,8,9], α-glucosidase inhibitory activities [10], a reduction in postprandial hyperglycemia [11] and reduction in cholesterol levels [12]. Tartary buckwheat grains are commonly milled to obtain flour, which is used as an ingredient in processed foods, such as soba noodles [13]. Tartary buckwheat bran, which contains episperm and embryo tissues, can also be obtained as a bio-residue of the buckwheat milling process [14]. This is commonly discarded as waste with accompanying environmental concerns, whereas it is in part used in low-value-added products [14]. It has been well-established that markedly higher levels of nutrient and functional components are included in Tartary buckwheat bran compared to its flour [13,14,15]. Thus, Tartary buckwheat bran is recognized as an important raw material with high levels of value-added components, such as rutin, for manufacturing health-beneficial foods [15]. Extracts from Tartary buckwheat bran have been reported to have beneficial effects on antioxidant activity and hyperlipidemia [16,17]. However, the utilization of Tartary buckwheat bran is hampered by its rough and bitter taste [18]. Thus, Tartary buckwheat bran needs to be processed to reduce or mask its bitterness [18]. The impact of the processing method, such as thermal and physical processing, on the quality of Tartary buckwheat bran has been investigated [18]. Tartary buckwheat bran has been used for wheat-based foods [19,20]. Tartary buckwheat seeds exhibit a high level of rutinosidase activity, which are capable of hydrolyzing rutin to quercetin and rutinose [21,22]. Recently, we have raised an epoch-making Tartary buckwheat cultivar, “Manten-Kirari”, with trace amounts of rutinosidase [23,24,25]. We have also carried out a research project regarding the high utilization of “Manten-Kirari” bran [26,27,28]. This present review aims to discuss the current knowledge, including our research results on the chemical composition, processing method, and food applications of Tartary buckwheat bran.

## 2. Nutritional Compounds of Tartary Buckwheat Bran

**Figure 1:**
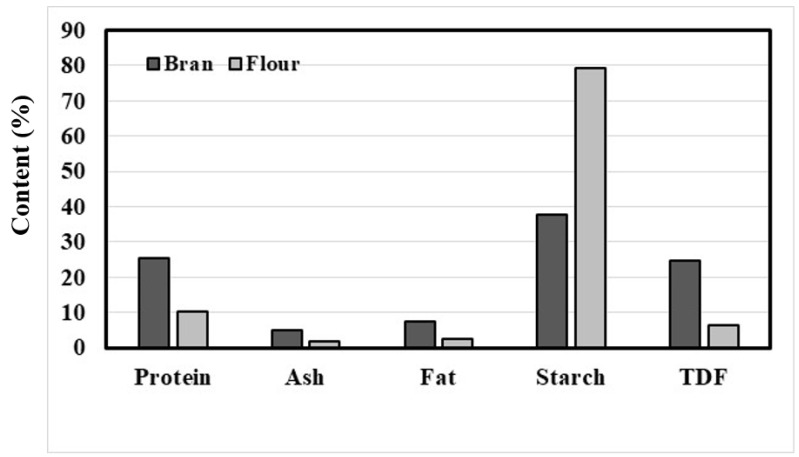
Chemical composition of Tartary buckwheat flour and bran. This graph is based on the data given by Bonafaccia et al. [13].

Tartary buckwheat bran is primarily composed of carbohydrates (starch, dietary fiber), protein, and fat. Bonafaccia et al. [13] compared the macronutrients of Tartary buckwheat flour and bran. Tartary buckwheat bran had higher levels of protein (25.3%), total dietary fiber (TDF) (24.8%), and fat (7.4%) and a lower level of starch (37.6%) than its flour (10.3% for protein, 6.3% for TDF, 2.5% for fat, 79.4% for starch) (Figure 1) [13]. Similar results were obtained in the two most recent reports of Sinkovič et al. [29,30], who studied the nutritional compounds of Tartary buckwheat milling fractions (hulls, bran, flour). Starch amounted to around 60% of the total dry weight of Tartary buckwheat groats [31]. Thus, starch is the major component not only of Tartary buckwheat flour but also of its bran [31]. Two homo-glucan biopolymers, amylose and amylopectin, are the main constituents of starch [32]. Amylopectin is a branched biopolymer composed of α-1,4-linked glucan chains connected by α-1,6-linked branches, while amylose is essentially a linear chain molecule composed almost completely of α-1,4-linked glucan chains [32]. Native starch granules generally contain 15–25% amylose and 75–85% amylopectin [32]. The physicochemical properties of starch are affected by their variation in amylose content [33,34]. The characteristics and structure of Tartary buckwheat starch have been studied extensively [35,36,37,38]. Tartary buckwheat starch granules are mainly from 2 to 14 µm in size and are generally polygonal in shape [36,37]. Similar to rice, wheat, and oat bran, Tartary buckwheat bran is also an important source of dietary fiber [13]. Dietary fiber (DF) is derived from plant cell walls, which are essentially composed of various polysaccharides, including cellulose, hemicellulose, pectic substances, and lignin [39]. DF cannot be digested and absorbed in the small intestine of humans but can be fermented in the large intestine [40]. It has been established that DF is a critical component with health benefits, such as the prevention of type 2 diabetes, obesity, and so on [41]. TDF can be classified generally into two major types based on water solubility: insoluble dietary fiber (IDF) and soluble dietary fiber (SDF) [42]. Bonafaccia et al. [13] reported that the proportion of SDF in the TDF of Tartary buckwheat bran was lower (4.77%) than that of its flour (8.27%). Wang et al. [43] examined the functional properties of a water-soluble polysaccharide fraction obtained from Tartary buckwheat. This investigation revealed that the polysaccharide fraction was composed mainly of 1,4-linked glucose. Moreover, the polysaccharide fraction displayed α-glucosidase inhibitory activity. Wu et al. [44] found that the SDF of Tartary buckwheat bran improved glucose and lipid metabolism in diabetic mice. Next to carbohydrates, proteins are the most abundant macronutrient of Tartary buckwheat bran. A Tartary buckwheat protein has a nutritionally balanced amino acid composition containing relatively high levels of arginine and lysine, which are limited in cereal proteins [45]. Moreover, Tartary buckwheat protein exhibits a relatively low digestibility [45] and shows health benefits, such as improving cholesterol metabolism in animals [46,47]. The development of Tartary buckwheat, a grain with higher levels of protein alongside an improved amino-acid composition, has been reviewed recently [48]. Fat is a relatively minor macronutrient of Tartary buckwheat, but it is concentrated in the bran fraction [13,29,30,49,50]. At least six fatty acids, including palmitic (C16:0), stearic (C18:0), oleic (C18:1), linoleic (C18:2), linolenic (C18:3), and arachidic (C20:0) acids, were found in Tartary buckwheat flour and bran [13,49,50,51]. Oleic, linoleic, and palmitic acids were the principal fatty acids of Tartary buckwheat [13,49,50,51].

Tartary buckwheat bran has high levels of micronutrients, such as minerals, free amino acids, and vitamins. Fourteen minerals were found in Tartary buckwheat milling fractions (flour, bran, hulls), and these minerals could be classified into two groups: major minerals (>0.2 g/kg DW) Mg, P, S, K, and Ca, and trace minerals (>0.01 mg/kg DW) Na, V, Cr, Mn, Fe, Co, Cu, Zn, and Mo [29,30]. K, P, Mg, S, Fe, Zn, Cu, Mo, and Co were concentrated in the bran fraction of Tartary buckwheat [29,30]. Bonafaccia et al. [52] analyzed the concentrations of Se, Cr, Rb, Zn, Fe, Co, Sb, Ba, Ni, Ag, Hg, and Sn in Tartary buckwheat flour and bran. The authors concluded that the concentrations of Se, Rb, Zn, Co, Ni, and Ag in bran were higher than those in flour. Peng et al. [51] examined the free amino acid composition in Tartary buckwheat flour and bran. The results revealed that Glu was the richest amino acid and that the concentration of detected amino acids in the bran was markedly higher than that in flour. As for vitamins, various types of vitamins, particularly B (B_1_, B_2_, and B_6_), C, and E (α-, γ-, and δ-tocopherols), were found in Tartary buckwheat [53,54]. Bonafaccia et al. [13] observed that Tartary buckwheat had higher contents of B vitamins than common buckwheat. They also found that the concentration of B vitamins was higher in Tartary buckwheat bran than in its flour. Dziedzic et al. [50] analyzed the tocopherol content in Tartary buckwheat milling fractions differing in particle size. They found that the bran fraction had the highest content of the total tocopherol.

## 3. Functional Compounds of Tartary Buckwheat Bran

Tartary buckwheat is described as an abundant source of functional compounds (i.e., polyphenolic compounds and D-*chiro*-inositol) [1,2]. Polyphenolic compounds have been associated with health-beneficial properties, such as antioxidant activity [55]. They can be classified as flavonoids, phenolic acids, coumarins, tannins, stilbenes, and so on [55]. Among polyphenols, flavonoids and phenolic acids from Tartary buckwheat have attracted considerable attention because of their unique composition and richness [2,14]. According to a study on the total flavonoid content of Tartary buckwheat (21 genotypes) and common buckwheat (18 genotypes), the measurement of the colorimetric method indicated that Tartary buckwheat flours contained nearly 10 times the total flavonoid content (6.65 to 22.74 mg/g DW) of common buckwheat flours (0.67 to 2.25 mg/g DW) [56]. Although bound polyphenols are included in popular cereal crops, such as rice, wheat, and maize, buckwheat predominantly contains free polyphenols. Guo et al. [57] found markedly higher concentrations of free flavonoids (17.19 to 30.14 μmol of rutin equivalent/g DW) than bound flavonoids (0.97 to 5.93 μmol of rutin equivalent/g DW) in two Tartary buckwheat varieties. The analysis by HPLC showed that, among flavonoids, flavonols, such as rutin, isoquercetin, quercetin, and kaempferol, were major components in Tartary buckwheat [58]. In a study conducted by Morishita et al. [4], rutin was the richest flavonoid, ranging in its content from 18.09 to 18.54 mg/g DW, and appeared to be the principal antioxidant in Tartary buckwheat grain. Another experiment indicated the free and bound rutin contents of 5.17 to 12.47 mg/g DW and 0.01 to 0.11 mg/g DW, respectively, in two Tartary buckwheat varieties [57]. It is well-known that polyphenolic compounds are concentrated in the bran fraction of Tartary buckwheat. Liu and Zhu [15] found that the total flavonoid concentration of Tartary buckwheat bran was much higher (7.16%) than that of its grains (2.42%). They also revealed that the major flavonoid in the bran fraction could be identified as rutin. Guo et al. [14] studied phenolic compositions and antioxidant properties in Tartary buckwheat milling fractions (hull, coarse bran, fine bran, and light flour). Their study observed that fine bran had the greatest total phenolic and flavonoid contents and ABTS scavenging activity. Most importantly, the fine bran possessed a much higher rutin content (74.31 mg/g DW) than any other fraction (2.88 to 4.86 mg/g DW). Concerning the phenolic acids determined by HPLC, the fine bran was rich in *p*-hydroxybenzoic (3.60 mg/g DW), caffeic (0.38 mg/g DW), chlorogenic (0.21 mg/g DW), and protocatechuic (0.18 mg/g DW) acids. Similarly, high rutin contents of 40.79 to 51.86 mg/g DW and 34.03 to 38.47 mg/g DW in Tartary buckwheat bran were observed by Peng et al. [51] and Sinkovič et al. [30], respectively. Peng et al. [51] also found that the quercetin content was 0.62 to 1.11 mg/g DW and the kaempferol content was only 0.02 to 0.04 mg/g DW. Thus, Tartary buckwheat bran is strongly recommended as a source of polyphenols, which have a wide variety of health benefits, particularly antioxidant properties. Wang et al. [16] investigated the effect of the phenolic extract of Tartary buckwheat bran on the antioxidant activity and lipid profile of hyperlipidemic rats. They showed that the extract significantly increased serum antioxidant activity and reduced blood and liver lipids. Another study revealed that the phenolic extracts of Tartary buckwheat bran had antioxidant capacities as well as antiproliferative activity on human liver cancer cells [17]. Additionally, Guo et al. [59] showed that the main phenolic antioxidants in the ethanol extract of Tartary buckwheat bran were quercetin and *p*-hydroxybenzoic acid. Wang et al. [60] revealed that the phenolic extract of the Tartary buckwheat bran possessed antibacterial activities against *Staphylococci aureus* (the family Staphylococcaceae and the order Bacillales)*, S. epidermidis*, and *Propionibacterium acnes* (the family Propionibacteriaceae and the order Propionibacteriales) as well as antioxidant activity. They also suggested that the antibacterial activities of Tartary buckwheat bran were potentially due to isoquercetin and quercetin or the mutual interaction between isoquercetin, quercetin, and rutin.

Besides polyphenolic compounds, D-*chiro*-inositol is also a bioactive compound present in Tartary buckwheat [1,2]. D-*chiro*-inositol has been known to have insulin-like bioactivity [61]. According to the reports of Steadman et al. [62] and Yang and Ren [63,64], the D-*chiro*-inositol content of Tartary buckwheat grain was 0.178 to 0.228 mg/g DW. It is well-known that most D-*chiro*-inositol exists in the form of its galactosyl derivatives, fagopyritols [62]. Common and Tartary buckwheat seeds have five different fagopyritols, with fagopyritol B1 being the major fagopyritol [62,65]. Steadman et al. [62] found that common buckwheat bran contained higher levels of D-*chiro*-inositol than its flour. Cheng et al. [66] studied the difference in the D-*chiro*-inositol content of different milled fractions of one common buckwheat variety and two Tartary buckwheat varieties. They found that D-*chiro*-inositol was concentrated in the bran fraction [66]. Kawa et al. [67] reported that the doses of a common buckwheat concentrate containing large amounts of D-*chiro*-inositol were effective at reducing serum glucose in streptozotocin-diabetic rats. Yao et al. [68] demonstrated that the oral administration of the Tartary buckwheat bran extract had a high D-*chiro*-inositol content and lowered plasma glucose in diabetic mice. Additionally, Hu et al. [69] demonstrated the beneficial effect of Tartary buckwheat extract containing high levels of D-*chiro*-inositol against hyperglycemia and hepatic steatosis in high fructose-treated mice.

## 4. Processing Method of Tartary Buckwheat Bran

To date, Tartary buckwheat bran is usually used as waste, resulting in environmental consequences [14]. As described in the previous section, Tartary buckwheat bran has high levels of functional compounds as well as nutrients [13,14]. Thus, the utilization of this bio-residue for making functional foods needs to be implemented immediately [15]. However, Tartary buckwheat bran has a strong rough, and bitter taste as well as large particle size, making it less feasible for use in the food industry [18]. Processing techniques, such as heat treatment and physical processing, have been proposed to reduce or mask the roughness and bitterness of Tartary buckwheat bran [18]. It is critical to understand the influence of the processing methods on the quality of Tartary buckwheat bran [70]. Ge and Wang [70] reported that thermal treatment at 180 °C for 30 min led to significant reductions in the contents of fatty acids, polysaccharides, amino acids, polyphenols, and total flavonoids in Tartary buckwheat bran. However, it was also found that rutin concentration decreased slightly from 33.29 to 31.77 mg/g DW with this treatment. Steam explosions (SE), where materials are heated and pressurized by a sudden decompression after direct contact with steam, is an economical and environmentally friendly processing method that is currently used when pretreating agricultural byproducts [71,72]. Li et al. [18] used SE to improve the roughness of Tartary buckwheat bran. They revealed that the contents of SDF and free and bound phenolics increased, and the TDF content decreased after SE. Another study by Li et al. [73] found that free quercetin content was enhanced by around 5 times, and the free rutin content was decreased by around 70% with SE pretreatment. Moreover, He et al. [74] demonstrated that SDF extracted from SE-modified Tartary buckwheat bran had a good hypoglycemic effect in type 2 diabetic *db*/*db* mice. A processing technique for reducing the particle size of Tartary buckwheat bran was also needed for extensive use. Xiao et al. [75] studied the influence of superfine grinding on the contents of phytochemical compounds of Tartary buckwheat bran and observed that the rutin content was enhanced significantly as the grinding time increased. These results were probably due to the fact that the release of phenolic compounds embedded in the cell wall matrix occurred during superfine grinding, as Zhu et al. [76] suggested. Xu et al. [77] examined the impact of different superfine grinding processes on the physicochemical properties of Tartary buckwheat bran. This study found that bran powder, when processed by wet grinding, had a markedly higher quercetin content than other samples, presumably because of the enzymatic conversion of rutin to quercetin during processing.

## 5. Food Uses of Tartary Buckwheat Bran

Although Tartary buckwheat bran has limited applications in food products, trials have been made to utilize it for wheat-based foods, such as noodles, bread, and cake. According to studies by Cho et al. [78] and Cho and Lee [79], extracts obtained from Tartary buckwheat bran were applied to wheat-based noodles for rutin fortification. Cho et al. [78] indicated that wheat-based noodles exhibited significantly increased antioxidant activities with the addition of the rutin-rich extract. Cho and Lee [79] observed that the rutin concentration in instant fried noodles did not alter largely due to their enhancement in a frying temperature from 150 to 190 °C. They also found that the addition of the extract did not lower the noodle quality attributes, such as oil uptake and mechanical properties. Zhang et al. [19] investigated the processing characteristics of a Tartary buckwheat bran–wheat flour blend and the influence of the weight proportion of Tartary buckwheat bran on the steamed bread quality. It was found that dough had the best kneading resistance when the weight proportion of Tartary buckwheat bran was 30%. Furthermore, the results of analyzing the steamed bread quality indicated that the specific volume decreased, and the hardness and chewiness increased gradually with the increase in the proportion of Tartary buckwheat bran. Xue et al. [20] evaluated the effect of the partial substitution of Tartary buckwheat flour with Tartary buckwheat bran on the in vitro starch digestibility of Tartary buckwheat-based dried noodles containing 30% wheat flour. They concluded that replacing Tartary buckwheat flour with Tartary buckwheat bran reduced the digestibility of the noodles. Heat-moisture treatment, which involves the treatment of starch granules at low moisture levels (10 to 30% moisture) for a period of time ranging from 15 min to 16 h and at high temperatures (90 to 120 °C) is a physical modification technology, which can alter the physicochemical and functional characteristics of starch and flour [80,81]. Research on the impact of heat-moisture treatment on the characteristics of Tartary buckwheat flour and starch has been performed to date [82,83]. More recently, Zhang et al. [84] applied Tartary buckwheat bran modified by heat-moisture treatment in steam bread making. They concluded that the steamed bread had similar qualities compared to the control wheat steam bread when the weight proportion of Tartary buckwheat bran was less than 20%. Pickering high internal phase emulsions (HIPEs) are generally defined as highly concentrated emulsions stabilized by solid particles with a dispersed phase volume fraction of >74% [85]. In a recent study conducted by Zhang et al. [86], sunflower oil-based Pickering HIPEs developed by Tartary buckwheat bran could partially replace butter for cake making.

Apart from wheat-based foods, Tartary buckwheat bran has been applied in the development of tea. The consumption of Tartary buckwheat tea has been traditionally recognized in China, and recently, its consumption has prevailed among other Asian countries and Europe because of its unique malty aroma [87]. Tartary buckwheat tea can be divided commonly into whole grain tea and whole plant tea (the mixture of stems, leaves, and flowers) [87]. The step of all kinds of Tartary buckwheat tea making should include heat treatment [87]. According to the report of Peng et al. [88], who analyzed the functional compounds in different types of Tartary buckwheat tea, whole bran tea and whole plant tea contained higher quercetin content but lower rutin content because of the action of rutinosidase, while converse results were found for whole grain tea. Li et al. [89] reported that there was no significant difference in the total phenolic content and antioxidant activity among the three types of Tartary buckwheat tea infusions, including the whole bran tea infusion.

## 6. Our Studies on Tartary Buckwheat Bran Using a Novel Cultivar ‘Manten-Kirari’

**Figure 2:**
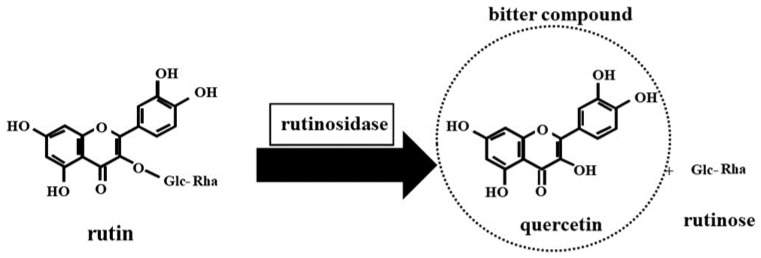
Rutin hydrolysis in Tartary buckwheat dough.

Tartary buckwheat seeds have markedly large amounts of rutinosidase, which can easily hydrolyze rutin into quercetin [21,22,90]. The milling of Tartary buckwheat grain and the mixing of flour and water causes the transformation of rutin to quercetin by rutinosidase [91,92,93]. As suggested by Kawakami et al. [94], quercetin and two unidentified compounds are the bitter compounds in Tartary buckwheat dough (Figure 2). The intense bitterness of Tartary buckwheat has hindered its high utilization [94]. Therefore, several heat treatments of Tartary buckwheat have been attempted to avoid the formation of bitter quercetin by deactivating rutinosidase [95,96,97,98]. Alternatively, our research team developed an epoch-making Tartary buckwheat cultivar, ‘Manten-Kirari’, which has trace-rutinosidase activity [23,24,25]. The rutinosidase activity in ‘Manten-Kirari’ is two orders of magnitude lower than that of conventional Tartary buckwheat cultivars with normal rutinosidase activity [24]. As shown by our previous investigations, the use of ‘Manten-Kirari’ flour enabled us to make rutin-rich foods with reduced bitterness [99,100,101]. However, even trace amounts of rutinosidase in ‘Manten-Kirari’ lead to the gradual enzymatic hydrolysis of rutin into quercetin during dough storage [24]. To overcome this, our research team found that the addition of sodium bicarbonate improved the residual ratio of rutin in the dough made from this cultivar [102]. Acute and subacute toxicity studies of rutin-rich dough from ‘Manten-Kirari’ were performed using rats [103]. The mutagenic activity of this cultivar was also evaluated in the Ames test [104]. These two investigations revealed that ‘Manten-Kirari’ flour was non-toxic. A clinical trial employing humans and rutin-rich noodles supplemented with ‘Manten-Kirari’ indicated that the intake of noodles resulted in body weight loss [105]. Another paper described that ‘Manten-Kirari’ could be discriminated from the major cultivars of Tartary and common buckwheat by a DNA marker [106]. Moreover, our research team evaluated preparation and thermal processing methods alongside the food use of Tartary buckwheat bran using the novel cultivar ‘Manten-Kirari’ [26,27,28].

First, we investigated the impact of the grain moisture content (6 to 22%) before roll milling on the rutin content of milling fractions of Tartary buckwheat ‘Manten-Kirari’ [26]. We revealed that operating the grain moisture content had an impact on the rutin content of flour and bran. Additionally, Tartary buckwheat bran was found to contain an extraordinarily high rutin content (65.00 to 85.00 mg/g DW) when the grain moisture content was 10 to 16%. Lower levels of rutin in Tartary buckwheat bran were included in the previous literature (Table 1). This was probably owing to the difference in the variety, growth environment, and/or the procedure of preparing the bran. Second, we were able to obtain roasted Tartary buckwheat ‘Manten-Kirari’ bran with a high rutin content, which has a high potential in the development of rutin-rich foods [27]. To prepare rutin-rich Tartary buckwheat bran, we examined the relationship of rutin content with color parameters (L*, a*, and b*) in different-sized Tartary buckwheat bran samples (20 to 100 g) during roasting treatment at different temperatures (160 °C to 240 °C) and at different times (10 to 30 min). Enhancing the roasting time and temperature decreased the rutin content and the values of L* and b*. Similar trends were found when reducing the sample amounts. In contrast, Tartary buckwheat bran still maintained high levels of rutin after roasting treatment for 10 min at <230 °C and for 20 min at 160 °C in sample amounts of 100 g. An increased rutin content has been closely linked to increased values of L* (lightness) and b* (yellowness). Thus, the rutin content in roasted Tartary buckwheat bran was predictable by measuring the color parameters using a Chroma Meter. Third, we determined the rutin content of roasted Tartary buckwheat bran and grain tea infusion samples for the development of a health-beneficial Tartary buckwheat tea [28]. Markedly higher concentrations of rutin were observed in tea infusion samples of roasted Tartary buckwheat bran than in those of roasted Tartary buckwheat grain. Therefore, roasted Tartary buckwheat bran could be more readily utilized as a functional ingredient in rutin-rich tea beverages through the utilization of a novel cultivar, ‘Manten-Kirari’, which has trace-rutinosidase activity.

## 7. Conclusions

To solve the environmental issue of the agri-food business, it is necessary to bring byproducts back to the food supply chain. The Tartary buckwheat flour-milling industry generates Tartary buckwheat bran, which is generally discarded, thus raising environmental concerns and reducing the total added value of Tartary buckwheat. Recently, Tartary buckwheat bran has gained the interest of researchers and the food and pharmaceutical industries because it possesses a range of nutrients and functional compounds. What is of great importance is that rutin, a common dietary flavonoid, is extremely concentrated in Tartary buckwheat bran. Because of its excellent chemical composition, Tartary buckwheat bran shows various health benefits, such as antioxidant lipid-lowering and antidiabetic properties. Several investigations have focused on the effective utilization of Tartary buckwheat bran as a functional ingredient in food products. There need to be more investigations to understand and reuse this potential bio-residue.

## Figures and Tables

**Table 1:** Results on the rutin content in various samples of Tartary buckwheat bran.

Rutin Content (mg/g DW)	Reference
65.00–85.00	Morishita et al. [26]
74.31	Guo et al. [14]
34.03–38.47	Sinkovič et al. [30]
40.79–51.86	Peng et al. [51]
33.29	Ge and Wang [70]
28.00	Xiao et al. [75]
51.70	Cho et al. [78]
36.10	Cho and Lee [79]
36.80	Oh et al. [98]

## Data Availability

Not applicable.
